# Appendicular Perforation Caused by a Fishbone: A Case Report

**DOI:** 10.7759/cureus.78362

**Published:** 2025-02-01

**Authors:** Sophia Bee Ting Tan, Heng-Chin Chiam

**Affiliations:** 1 General Surgery, Cairns Hospital, Queensland, AUS

**Keywords:** acute appendicitis diagnosis, atypical appendicitis, fishbone perforation, foreign bodies, laparascopic surgery

## Abstract

Foreign body ingestion is a common clinical occurrence, with most objects passing through the gastrointestinal tract uneventfully. However, sharp foreign bodies, such as fishbones, pose a significant risk for complications, including perforation and appendicitis. We present the case of a 48-year-old man who presented with a one-week history of progressively worsening lower abdominal and right lower quadrant pain. Computed tomography (CT) imaging confirmed acute uncomplicated appendicitis with a suspected foreign body. Further history revealed the recent consumption of Barramundi fish. The patient underwent laparoscopic appendectomy, which identified a fishbone perforating the appendiceal wall. The procedure was successfully completed without complications, and the patient had an uneventful recovery with discharge the following day. This case highlights the importance of considering foreign body ingestion as a potential cause of appendicitis, particularly in patients with relevant dietary history. Prompt diagnosis and surgical intervention are crucial in managing such cases effectively.

## Introduction

Foreign body ingestion is common, with most objects passing naturally without complications; however, sharp objects such as fishbones can cause gastrointestinal injuries, including perforation and appendicitis. Foreign bodies in the appendix are rare, with an incidence of 0.005% to 0.113%, and fishbone-induced appendicitis is even rarer, estimated at less than 0.0005% [1-4]. Due to the appendix’s limited motility, sharp objects like fishbones may become lodged, leading to inflammation and perforation. While expectant management is standard for most ingested foreign bodies, sharp objects may necessitate surgical intervention [5]. We present a rare case of perforated appendicitis secondary to fishbone ingestion in a 48-year-old male diagnosed preoperatively through imaging and confirmed intraoperatively.

## Case presentation

A 48-year-old man presented to the emergency department with a one-week history of progressively worsening lower abdominal and right lower quadrant pain. Physical examination revealed mild tenderness in the suprapubic and right iliac fossa regions, with stable vital signs.

Laboratory investigations showed mildly elevated white cell count (WCC) and C-reactive protein (CRP) levels (Table 1).

**Table 1:** The patient's laboratory parameters on admission

Laboratory parameters	Level on admission	Normal range
White cell count (WCC)	11.9x10^9/L	4.5-11.0×10^9/L
C-reactive protein (CRP)	6.5 mg/L	<0.5 mg/L

**Figure 1:**
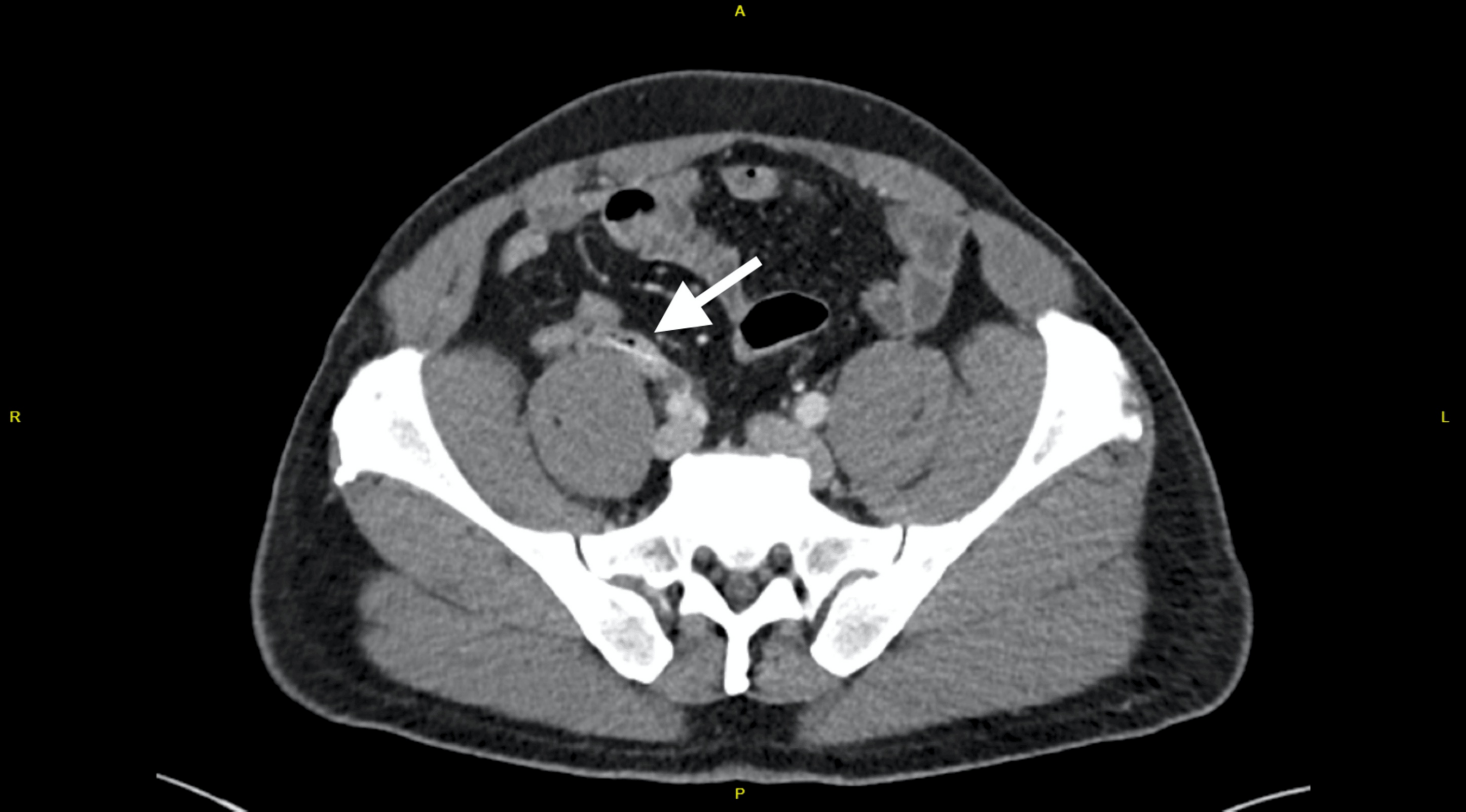
Axial contrast-enhanced CT scan of the abdomen revealed a dilated appendix with wall thickening indicative of acute appendicitis, along with a 2 cm linear hyperdense structure (white arrow).

Computed tomography (CT) imaging confirmed acute uncomplicated appendicitis with a 20 mm linear opacity, indicative of an ingested foreign body (Figure 1). Upon further questioning, the patient recalled consuming Barramundi fish two weeks earlier.

**Figure 2:**
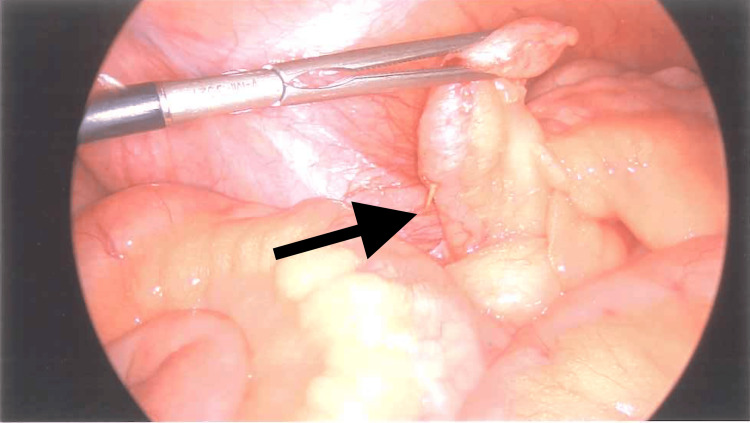
Laparoscopic image of a foreign body, namely, the fishbone (black arrow), which induced perforated appendicitis.

**Figure 3:**
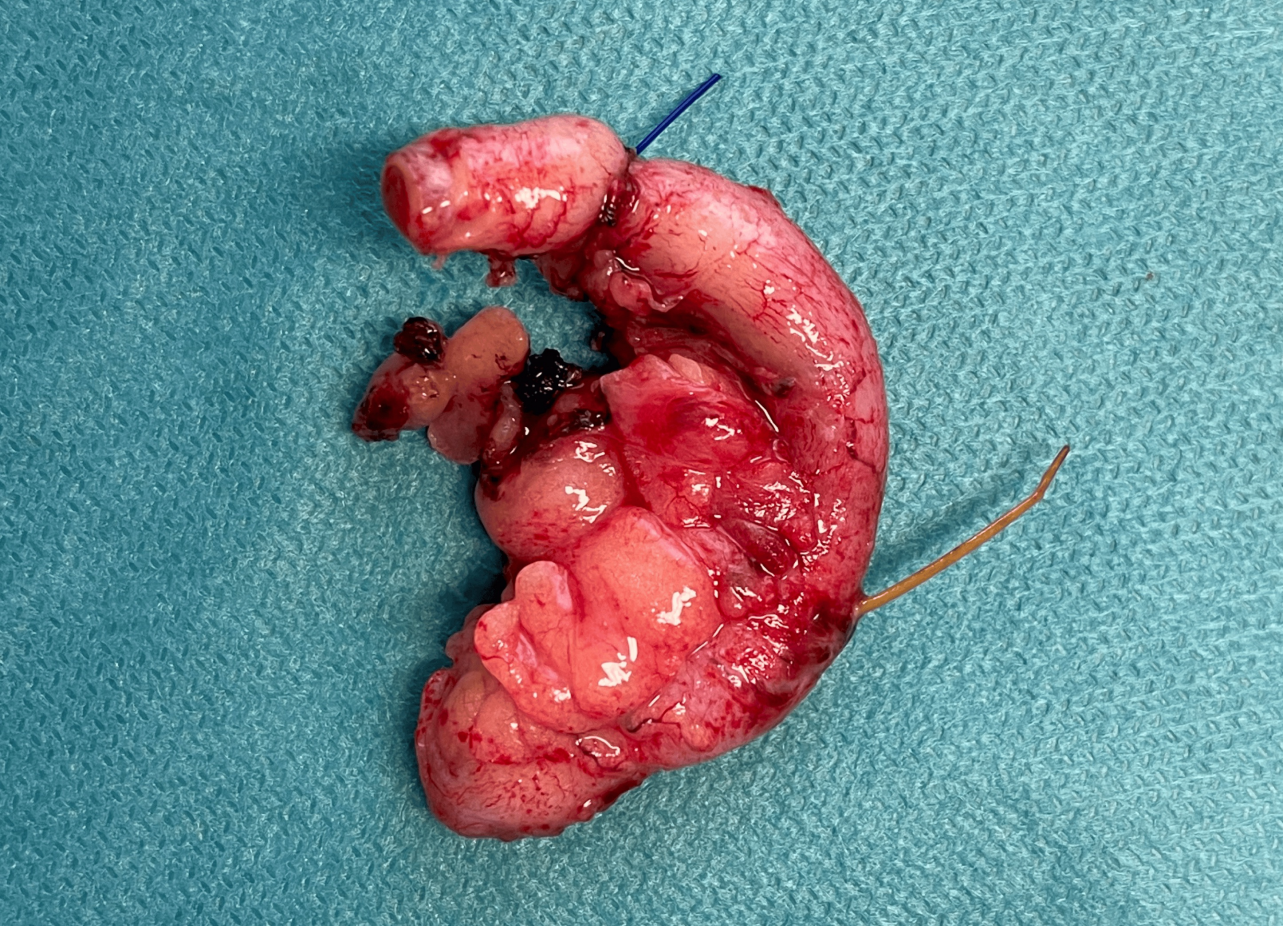
Photograph of the surgical specimen showed appendicitis due to a fishbone.

The patient was commenced on intravenous antibiotics, analgesia, and antiemetics. A laparoscopic appendectomy was performed, during which a fishbone was discovered perforating the proximal one-third of the appendiceal wall with a surrounding omental mass (Figure 2 and Figure 3). The procedure was completed without complications. The patient had an uneventful postoperative course and was discharged the following day with appropriate follow-up arrangements. The histopathology results indicate a localized reactive inflammatory response to foreign material in the appendix.

## Discussion

Fishbone-induced appendicitis is an exceedingly rare but significant clinical entity that warrants attention due to its potential for serious complications. Although foreign body ingestion is relatively common, the vast majority of ingested objects pass harmlessly through the gastrointestinal tract without incident. However, in rare cases, such as this one, a foreign body can become lodged in the appendix, leading to inflammation and perforation. This case highlights the diagnostic and therapeutic challenges associated with fishbone-induced appendicitis and underscores the importance of early recognition and prompt surgical intervention.

Foreign body-induced appendicitis is a rare condition, with an incidence ranging from 0.005% to 0.113% in large case series [1-4]. Studies conducted in the USA have reported very few cases, largely due to differences in dietary habits and food processing methods compared to East Asian populations, where fish consumption is higher [5]. The appendix's gravity-dependent location and weak peristaltic movement contribute to the retention of ingested foreign bodies, making it difficult for such objects to exit the lumen once they enter [6].

The diagnosis of fishbone-induced appendicitis is often challenging due to the nonspecific nature of clinical symptoms and the difficulty in detecting fishbones in imaging studies. Symptoms can mimic common conditions such as cholecystitis, bowel obstruction, and even malignancy.

Abdominal radiography has a low sensitivity (32%) for detecting fishbones, making CT the preferred imaging modality [7]. Computed tomography scans provide high sensitivity (100%) and specificity (97.8%) in detecting ingested foreign objects [4,7]. Goh et al. reported that the sensitivity of CT in detecting intra-abdominal fishbones was 71.4% (five out of seven cases) on initial interpretation, which increased to 100% (seven out of seven cases) upon retrospective review of the CT images. Characteristic CT findings may include intestinal wall thickening, localized pneumoperitoneum, regional fat stranding, and signs of intestinal obstruction. However, these features are non-specific, and definitive diagnosis typically depends on the direct visualization of the calcified fishbone on imaging [8]. Given the potential for subtle presentations, careful evaluation by the radiologist is necessary, as fishbones can be subtle and easily overlooked.

The presence of a foreign body in the appendix, particularly sharp objects such as fishbones, poses a high risk of perforation and abscess formation. In a systematic review of case reports by Elmansi Abdalla et al., 91% of cases of foreign body-induced appendicitis were managed surgically. Non-surgical approaches, such as bowel preparation, endoscopic procedures, intravenous antibiotics, laxatives, and serial imaging, were attempted but were unsuccessful in patients who ultimately required surgery. Only 9% of cases were successfully managed non-surgically, with positive outcomes achieved through conservative treatment and colonoscopy [9].

Laparoscopic appendectomy is a safe and effective approach, allowing for direct visualization and removal of the foreign body while minimizing surgical trauma. In this case, the patient underwent laparoscopic appendectomy with successful removal of the fishbone and surrounding omental mass, with an uneventful postoperative recovery. Open appendectomy may still be considered in cases with extensive abscess formation or when laparoscopy is not feasible.

Given the severity of fishbone-induced appendicitis, prevention should focus on patient education, especially in populations with high fish consumption. A prospective study in Singapore by Arulanandam et al. identified risk factors such as using chopsticks or cutlery, deboning fish in the mouth, and wearing dentures while eating fish [10]. Addressing these modifiable behaviors through education may help prevent serious complications from fishbone ingestion.

## Conclusions

This case report underscores the importance of considering foreign body ingestion as a potential cause of appendicitis, especially in patients with a history of recent food consumption that may include fish or other bone-containing foods. Early recognition, accurate imaging, and prompt surgical intervention are crucial in ensuring favorable outcomes. As imaging techniques continue to advance, improved detection rates may facilitate earlier diagnosis and management of such rare but potentially serious cases.
